# Clinical outcomes of Interleukin-2 therapy in advanced cancer: meta-analysis of over 62 trials

**DOI:** 10.1186/2051-1426-1-S1-P149

**Published:** 2013-11-07

**Authors:** Brendon J Coventry, Martin L Ashdown, Richard Bright

**Affiliations:** 1 Surgery, University of Adelaide, Adelaide, SA, Australia; 2 Medicine, University of Melbourne, Melbourne, VIC, Australia

## Introduction

Interleukin-2 (IL2) therapy for cancer has stood the test of time, with continued widespread use with clinical complete responses (CR) variably reported at 5%-20% for advanced malignant melanoma, renal cell carcinoma, and a range of other cancers. IL-2 is recognized to be curative with strong durable long-term 5-10-year survivals, usually resulting from CR’s.

## Methods

The literature was reviewed for clinical trials using low, intermediate and high dose IL2 therapy, alone or in combination. A meta-analysis was performed on the data.

## Results

At completion, 62 trials were identified as meeting the inclusion criteria, encompassing 5312 patients treated using IL2. A complete response rate of 6% across the entire IL2 treated population was determined. IL2 alone and in combination, and the dosing was investigated. All doses and regimens were capable of producing CR’s.

## Conclusions

Finally, after over 20 years of use and investigation of IL2, the mechanisms of action of IL2 as a ‘homeostatic’ cytokine are becoming clearer. The physiological feedback of IL2 actions over T-cell effector and regulatory functions indicate that there is a homeostatic balance, which is capable of being manipulated by exogenous (therapeutic) IL-2 towards either net responsiveness or tolerance. This is underpinned by transient expression of IL-2 receptor levels on the respective effector or regulatory populations. In this regard, the timing and frequency of IL2 administration will necessarily determine the clinical outcome through ‘immune synchronization’. The way forward is to monitor systemic inflammation, to determine the optimal time for IL2 dose delivery; and not to give up on the patient too early because non-responsiveness can be turned into responsiveness with repetitive accurate dosing. This phenomenon is just becoming apparent with other therapies, showing that the solution to the problem is obtainable by carefully coordinated and timed therapeutic administration.

